# Genetic Polymorphisms of *TGFB1*, *TGFBR1*, *SNAI1* and *TWIST1* Are Associated with Endometrial Cancer Susceptibility in Chinese Han Women

**DOI:** 10.1371/journal.pone.0155270

**Published:** 2016-05-12

**Authors:** Li Yang, Ya-Jun Wang, Li-Yuan Zheng, Yu-Mian Jia, Yi-Lin Chen, Lan Chen, Dong-Ge Liu, Xiang-Hong Li, Hong-Yan Guo, Ying-Li Sun, Xin-Xia Tian, Wei-Gang Fang

**Affiliations:** 1 Department of Pathology, Key Laboratory of Carcinogenesis and Translational Research (Ministry of Education), Peking University Health Science Center, Beijing, China; 2 Department of Pathology, Peking University Third Hospital, Beijing, China; 3 Department of Pathology, Beijing Hospital, Beijing, China; 4 Department of Pathology, Peking University School of Oncology, Beijing Cancer Hospital & Institute, Beijing, China; 5 Department of Gynecology, Peking University Third Hospital, Beijing, China; 6 Key Laboratory of Genomic and Precision Medicine, Chinese Academy of Sciences, Beijing, China; Children's National Medical Center, Washington, UNITED STATES

## Abstract

Endometrial cancer (EC) is a complex disease involving multiple gene-gene and gene–environment interactions. TGF-β signaling plays pivotal roles in EC development. This study aimed to investigate whether the genetic polymorphisms of TGF-β signaling related genes *TGFB1*, *TGFBR1*, *SNAI1* and *TWIST1* contribute to EC susceptibility. Using the TaqMan Genotyping Assay, 19 tagging-SNPs of these four genes were genotyped in 516 EC cases and 707 controls among Chinese Han women. Logistic regression (LR) showed that the genetic variants of *TGFB1* rs1800469, *TGFBR1* rs6478974 and rs10733710, *TWIST1* rs4721745 were associated with decreased EC risk, and these four loci showed a dose-dependent effect (*P*trend < 0.0001). Classification and regression tree (CART) demonstrated that women carrying both the genotypes of *TGFBR1* rs6478974 TT and rs10512263 TC/CC had the highest risk of EC (aOR = 7.86, 95% CI = 3.42–18.07, *P*<0.0001). Multifactor dimensionality reduction (MDR) revealed that *TGFB1* rs1800469 plus *TGFBR1* rs6478974 was the best interactional model to detect EC risk. LR, CART and MDR all revealed that *TGFBR1* rs6478974 was the most important protective locus for EC. In haplotype association study, *TGFBR1* haplotype CACGA carrier showed the lowest EC risk among women with longer menarche-first full term pregnancy intervals (˃11 years) and BMI˂24 (aOR = 0.39, 95% CI = 0.17–0.90, *P* = 0.0275). These results suggest that polymorphisms in *TGFB1*, *TGFBR1*, *SNAI1* and *TWIST1* may modulate EC susceptibility, both separately and corporately.

## Introduction

Endometrial cancer (EC) is one of the most common gynecological malignancies worldwide. According to the National Central Cancer Registry of China, the incidence of EC was about 18.5 per 100,000 urban women in 2011 [1]. Longer lifetime estrogen exposure such as early menarche, late menopause, nulliparity and postmenopausal estrogen use, is related with increased EC risk, which indicates that estrogen can drive endometrial carcinogenesis. Traditionally, there are three subtypes of EC distinguished by biological and clinical courses: hormonally driven Type I with endometrioid histology, Type II with non-endometrioid serous or clear cells, and familial aggregated EC [2]. The increasing EC prevalence in recent years highlights the importance for developing strategies for its risk estimation and prevention [1].

It’s well known that the genetic variants such as single nucleotide polymorphisms (SNPs) play important roles in cancer susceptibility. The contributions of genetic variations or mutations to cancer risk in a population depend on their frequency and penetrance [3]. Although the high-penetrant and low-frequent mutations such as *TP53*, *PTEN* confer high risk to rare familial aggregated EC [4, 5], the vast majority of EC are sporadic and involve polygenes, indicating that the common polymorphisms play predominant roles in carcinogenesis because of their high frequency [4].

Genome-wide association study (GWAS) still remains costly, so many association studies on SNPs with EC risk have been performed in the context of candidate genes, including genes regulating DNA damage repair, steroid and carcinogen metabolism, cell-cycle control and apoptosis [2]. The epithelial-to-mesenchymal transition (EMT), a crucial process in tumor progression, promotes tumor cell invasion from the primary foci to surrounding tissues. To date, many molecules have been validated to trigger epithelial dedifferentiation and EMT, such as those involved in TGF-β signaling as well as EMT-related transcriptional factors Snail and Twist [6, 7]. Canonical mediation of TGF-β1 (encoded by *TGFB1*) signaling is via TβRI (encoded by *TGFBR1*) and TβRII to form SMAD transcriptional complexes, thus leading to the rapid activation of the transcriptional factors Snail and Twist (encoded by *SNAI1* and *TWIST1*) [8, 9].

Germline mutations in signaling components of TGF-β family have been described to result in malignancies along with other heritable disorders. Polymorphism association studies in genes of this signaling pathway have been mainly focused on the risk of breast cancer [10–12], ovarian cancer [13] or colorectal cancer [14]. Until now, there have been few studies to explore the association of germline variants in TGF-β related genes with EC among Chinese Han population. We hypothesized that common genetic polymorphisms of *TGFB1*, *TGFBR1*, *SNAI1* and *TWIST1* may influence EC susceptibility in Chinese Han women.

## Materials and Methods

### Ethics statement

This study was approved by the Peking University IRB (reference no. IRB00001052-11029). Written consents were obtained from all control samples. EC patient’s genomic DNAs were extracted from archived formalin-fixed paraffin-embedded normal fallopian tube tissues. Because the contact information of EC patients who were treated in the hospitals before 2011 was not clear, PKU IRB approved our application to waive informed consent for the archived EC samples collected before April 2011. This study only used this part of samples. All the data/samples were used anonymously.

### Study population

A total of 516 cases with pathological diagnosed endometrial adenocarcinoma were recruited from Peking University Third Hospital, Beijing Cancer Hospital and Beijing Hospital between 1999 and 2011. Patients with history of cancer, metastasized cancer from other organs, and radiotherapy or chemotherapy history were excluded from our study. The epidemiological information including age, body mass index (BMI), age at menarche/menopause/primiparity, smoking history and family history of cancer in the first-degree relatives was collected. The eligible 707 controls were randomly selected from women who participated in a community-based screening program for non-infectious diseases conducted in Beijing between 2011 and 2012. The selection criteria included no history of cancer, Chinese Han ethnic background and frequency-matched to the cases by 5 year-age. All controls provided the same epidemiological information as that we collected from the cases. The characteristics of the 707 controls and the 516 cases are summarized in S1 Table. This study was approved by the Ethics Committee of Peking University Health Science Center.

### SNPs selection

We selected tagging-SNPs (tSNPs) by using Haploview v.4.2 software program based upon Chinese Beijing population (CHB) data from HapMap Project phase I, II and III merged database (http://hapmap.ncbi.nlm.nih.gov/). All tSNPs with a minor allele frequency (MAF) ≥5% were identified and partitioned in bins according to the r^2^ linkage disequilibrium (LD) statistic (threshold ≥0.8). A maximally informative tSNP was then selected from each bin, and these tSNPs could capture all known common genetic variants within the entire gene [15]. For *TGFB1*, seven tSNPs spanning 5 kb to each flank were identified, these being rs1800469, rs2241716, rs4803455, rs747857, rs12983047, rs10417924 and rs12981053. For *TGFBR1*, the minimum set of five tSNPs, rs10988706, rs6478974, rs10512263, rs10733710 and rs334348, ranging from 5 kb upstream to 5 kb downstream, was chosen. For *SNAI1*, three tSNPs spanning 2kb to each flank were selected, these being rs6125849, rs4647959 and rs6020178. For *TWIST1*, four common tSNPs covering 2kb flanking sequence were identified, these being rs2285682, rs2285681, rs4721746 and rs4721745.

### DNA isolation and genotyping assay

Genomic DNA for controls was isolated from peripheral blood leukocytes, whereas cases’ genomic DNA was extracted from formalin-fixed paraffin-embedded normal fallopian tube tissues. Genotyping was conducted with the ABI 7900HT^®^ Real-Time PCR System (Applied Biosystems, Foster City, California) using TaqMan^®^ Assay in compliance with the manufacturer’s instructions. Primers and probes (FAM- and VIC- labeled) were supplied by ABI incorporation and the PCR reaction system was the same as described previously [16]. Briefly, all assays were carried out in 384-well plates with negative and positive controls. Plates were sealed and heated at 95°C for 5min, then subjected to 45–50 cycles of 92°C for 15s and 60°C for 1min. Data from plates failing in more than 15% samples were excluded from the analysis. At least 1% of the samples were duplicated randomly in each SNP genotyping assay, and the concordance between duplicates was more than 99%.

### Statistical analysis

Differences in the distribution of demographic characteristics and selected variables between controls and cases were calculated by two-sided Pearson’s χ^2^ test or Student’s t test, where appropriate. Hardy-Weinberg equilibrium (HWE) was evaluated in controls using goodness-of-fit χ^2^ test within each tSNPs. The D’ values of LD plots were produced using the Haploview program. The expectation–maximization (EM) algorithm was used to evaluate the most probable haplotype by maximum-likelihood estimation among current population. A two-sided χ^2^ test was employed to compare differences in the distribution of genotypes and alleles between cases and controls. Each genotype was assessed in terms of additive (co-dominant), dominant and recessive models of inheritance. Also, Cochran-Armitage trend test was performed to estimate the association between EC risk and allele dose in each tSNP (*P* trend). Odds ratios (OR) and 95% confidence intervals (CI) were assessed by using univariate and multivariate unconditional logistic regression (LR), with adjustment for BMI, age at menarche/primiparity, menopause status, number of childbearing and family history of cancer. Statistical significance was defined as *P*<0.05. A Bonferroni-corrected *P* value was carried out in individual tSNPs and haplotype/diplotype association analysis. The potential gene-environment interactions between *TGFBR1* haplotype CACGA and clinical risk factors (estrogen exposure, family history of cancer and BMI) were assessed by LR in stratified population. All statistics were analyzed by SAS software (v.9.1; SAS Institute, Cary, NC).

Classification and regression tree (CART) analysis was performed for high-order gene-gene interactions using SPSS software (v.19.0; SPSS Inc., Chicago, IL, USA) to build a decision tree via recursive partitioning. The decision tree started with a root node which contained the total sample and split into two child nodes. The splitting process continued until the terminal nodes had no subsequent statistically significant splits or reached a pre-supposed minimum size, and then the terminal subgroups were further analyzed. The case rate was calculated for each terminal node and the association of subgroups with EC risk was evaluated by LR analysis, using the subgroup with the least percentage of cases as reference. The ORs and 95% CI were adjusted as mentioned above.

Multifactor dimensionality reduction (MDR) analysis was performed to identify high-order interaction models that were associated with EC risk using open-source MDR software (v.2.0 beta 8.4, http://www.epistasis.org) [17]. Statistical significance was determined using permutation testing in MDRpt (v.1.0 beta 2.0). MDR analysis collapsed multi-dimensional data into a single independent dimensional variable with two levels (high and low risk) using the ratio of the number of cases to the number of controls, and thereby reduced multiple dimensional data into one dimension and permitted detection of interactions in relatively small sample sizes. The new one-dimensional multi-locus genotype variable was evaluated for its ability to classify and examine disease status through cross-validation and permutation test. The best candidate interaction model was regarded as the one with maximal testing accuracy and cross-validation consistency (CVC). To better confirm and visualize the interaction models, we further built an entropy-based interaction dendrogram. This would enable the loci that strongly interact to each other to appear close together at the branches of the tree, and those with weak interaction to appear distant from one another. MDR 1,000-fold permutation results were regarded as statistically significant at *P*<0.05. The conjoint effect of the variables in the best model was assessed by LR analysis.

## Results

### Characteristics of study population

The characteristics of population were herein described in S1 Table. The controls and cases seemed to be adequately matched on age (*P* = 0.7528). The cases, as expected, had higher BMI (*P***<**0.0001), earlier age of menarche (*P***<**0.0001) and later age of menopause (*P* = 0.0002) compared with the controls. In addition, the percentage of nulliparous women in patients was significantly higher than that in controls (*P*<0.0001). EC patients were more likely to have family history of cancer in first-degree relatives (*P* = 0.0464). These variables with significant differences between cases and controls were used in multivariate LR models to further adjust for any possible confounding effect on the association of selected genetic variants with risk of EC.

### LD degree between tSNPs

**Fig 1:**
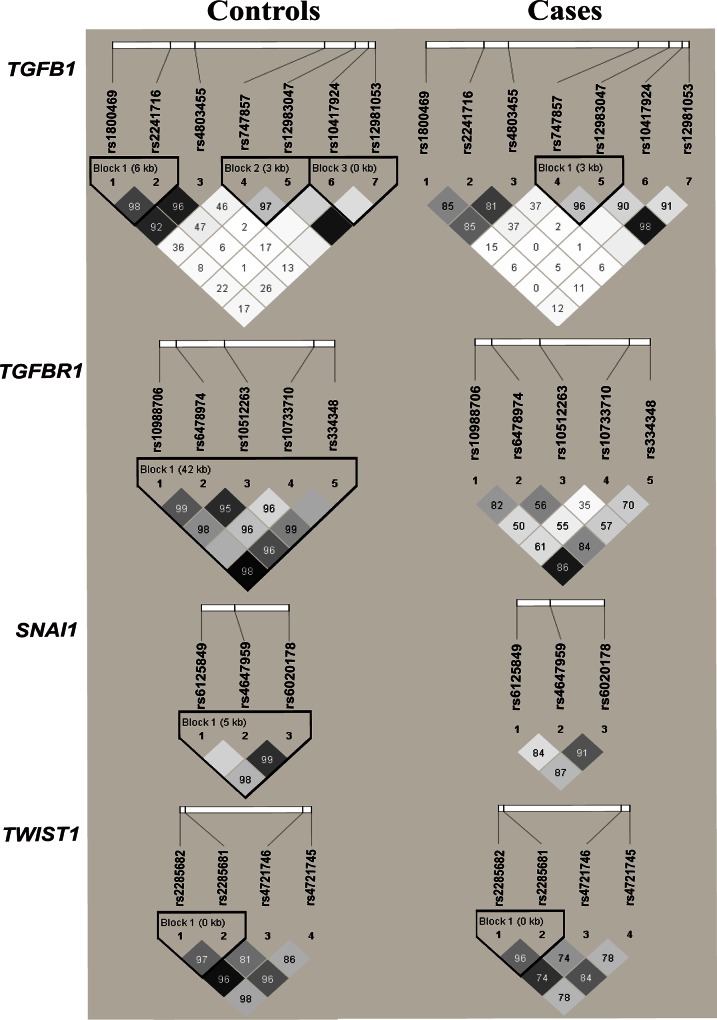
LD maps of the analyzed 19 tSNPs in controls and cases. The value in each diamond indicates pairwise LD between tSNPs (measured as D’× 100, 10 means 0.10, 1 means 0.01). The shading with a dark grey-to-white gradient reflects higher to lower LD values.

The genotype frequencies for selected 19 tSNPs were all consistently in agreement with those expected by HWE in controls (*P*˃0.05, S2 Table). For our study, haplotype blocks were reconstructed in cases and controls as well as in HapMap CHB population based on D’ value (Fig 1). There were some differences in SNPs’ pairwise LD between controls and cases. For *TGFB1*, three LD blocks were reconstructed in disease-free participants. For *TGFBR1* as well as *SNAI1*, all selected tSNPs were reconstructed into one high-LD block in controls. For *TWIST1*, only one haplotype block was reconstructed, in which the rs4721746 and rs4721745 were excluded from the analysis because their MAFs were lower than 5%.

### Association of individual tSNPs in *TGFB1*, *TGFBR1*, *SNAI1*, *TWIST1* with EC risk by LR analysis

As shown in Table 1, two-sided χ^2^ test indicated statistically differences of genotype frequencies between cases and controls in polymorphisms *TGFB1* rs1800469 (C˃T), *TGFBR1* rs6478974 (T˃A), *TGFBR1* rs10512263 (T˃C), *TGFBR1* rs10733710 (G˃A), *TWIST1* rs4721746 (C˃A) and *TWIST1* rs4721745 (C˃G) (*P***<**0.0001, **<**0.0001, *P* = 0.0059, 0.0016, 0.0045 and 0.0430, respectively). Also, multivariate LR showed that *TGFB1* rs1800469, *TGFBR1* rs6478974 and rs10733710, and *TWIST1* rs4721745 were protective loci for EC susceptibility under dominant or recessive models, whereas *TWIST1* rs4721746 was a risk locus (Table 1). *TGFBR1* rs6478974 remains significant under an additive and dominant models after applying the stringent Bonferroni correction (Bonferroni-corrected *P***<**0.05). Other tSNPs did not show statistical significance in the multivariate analysis (S3 Table).

**Table 1:** Univariate and multivariate analysis of the association of candidate tSNPs with EC risk.

Gene	SNPs	Genotype	Cases (%)	Controls (%)	*P*^a^	*P*^b^	*P*_trend_ (*P*^d^)	OR (95% CI)	*P*	aOR (95% CI)^c^	*P*^c^ (*P*^d^)
*TGFB1*	rs1800469	CC	189 (36.63)	170 (24.05)	**<0.0001**		**0.0111**(0.2109)	Reference		Reference	
		CT	197 (38.18)	372 (52.62)				0.48 (0.36–0.62)	**<0.0001**	0.57 (0.41–0.80)	**0.0012 (0.0456)(0.0228)**
		TT	130 (25.19)	165 (23.34)				0.71 (0.52–0.97)	**0.0292**	0.86 (0.59–1.27)	0.4463
		T allele frequency	457 (44.28)	702 (49.65)		**0.0087**					
		CT/TT vs. CC (dominant model)						0.55 (0.43–0.70)	**<0.0001**	0.66 (0.48–0.90)	**0.0093** (0.1767)
		TT vs. CC/CT (recessive model)						1.11 (0.85–1.44)	0.4538	1.27 (0.92–1.75)	0.1454
*TGFBR1*	rs6478974	TT	282 (54.65)	286 (40.45)	**<0.0001**		**0.0006(0.0114)**	Reference		Reference	
		TA	163 (31.59)	326 (46.11)				0.51 (0.40–0.65)	**<0.0001**	0.55 (0.40–0.75)	**0.0001 (0.0038)**
		AA	71 (13.76)	95 (13.44)				0.76 (0.54–1.07)	0.1193	0.86 (0. 57–1.32)	0.4903
		A allele frequency	305 (29.55)	516 (36.49)		**0.0003**					
		TA/AA vs. TT (dominant model)						0.56 (0.45–0.71)	**<0.0001**	0.63 (0.47–0.83)	**0.0010 (0.0190)**
		AA vs. TT/TA (recessive model)						1.03 (0.74–1.43)	0.8706	1.13 (0.76–1.69)	0.5467
*TGFBR1*	rs10512263	TT	298 (57.75)	379 (53.61)	**0.0059**		0.8535	Reference		Reference	
		TC	168 (32.56)	284 (40.17)				0.75 (0.59–0.96)	**0.0221**	0.85 (0.63–1.14)	0.2693
		CC	50 (9.69)	44 (6.22)				1.45 (0.94–2.23)	0.0952	1.44 (0.86–2.42)	0.1631
		C allele frequency	268 (25.97)	372 (26.31)		0.8504					
		TC/CC vs. TT (dominant model)						0.85 (0.67–1.06)	0.1500	0.93 (0.70–1.23)	0.6240
		CC vs. TT/TC (recessive model)						1.62 (1.06–2.47)	**0.0257**	1.54 (0.93–2.55)	0.0914
*TGFBR1*	rs10733710	GG	359 (69.57)	471 (66.62)	**0.0016**		0.8505	Reference		Reference	
		GA	121 (23.45)	212 (29.99)	** **			0.75 (0.58–0.97)	**0.0306**	0.59 (0.42–0.82)	**0.0018** (0.0684)
		AA	36 (6.98)	24 (3.39)				1.97 (1.15–3.36)	**0.0130**	1.48 (0.79–2.79)	0.2206
		A allele frequency	193 (18.70)	260 (18.39)		0.8435					
		GA/AA vs. GG (dominant model)						0.87 (0.68–1.11)	0.2747	0.69 (0.51–0.94)	**0.0176** (0.3344)
		AA vs. GG/GA (recessive model)						2.13 (1.26–3.62)	**0.0050**	1.78 (0.96–3.32)	0.0694
*TWIST1*	rs4721746	CC	399 (77.33)	512 (72.42)	**0.0045**		0.3457	Reference		Reference	
		CA	92 (17.83)	175 (24.75)				0.68 (0.51–0.90)	**0.0067**	0.70 (0.49–0.99)	**0.0432** (1.6416)
		AA	25 (4.84)	20 (2.83)				1.60 (0.88–2.93)	0.1242	2.11 (1.05–4.22)	**0.0354** (1.3452)
		A allele frequency	142 (13.77)	215 (15.21)		0.3220					
		CA/AA vs. CC (dominant model)						0.77 (0.59–1.00)	0.0522	0.85 (0.61–1.17)	0.3061
		AA vs. CC/CA (recessive model)						1.75 (0.96–3.19)	0.0675	2.29 (1.15–4.57)	**0.0188** (0.3572)
*TWIST1*	rs4721745	CC	193 (37.40)	216 (30.55)	**0.0430**		**0.0343**(0.6517)	Reference		Reference	
		CG	231 (44.77)	352 (49.79)				0.73 (0.57–0.95)	**0.0179**	0.82 (0.60–1.13)	0.2246
		GG	92 (17.83)	139 (19.66)				0.74 (0.53–1.03)	0.0723	0.60 (0.40–0.92)	**0.0173** (0.6574)
		G allele frequency	415 (40.25)	630 (44.55)		**0.0337**					
		CG/GG vs. CC (dominant model)						0.74 (0.58–0.94)	**0.0123**	0.76 (0.56–1.02)	0.0642
		GG vs. CC/CG (recessive model)						0.89 (0.66–1.19)	0.4193	0.68 (0.46–0.98)	**0.0401** (0.7619)

tSNPs, tagging single nucleotide polymorphisms; EC, endometrial cancer; OR, odds ratios; CI, confidence intervals.

^a^ Two-sided χ2 test for difference in frequency distribution of genotypes between cases and controls.

^b^ Two-sided χ2 test for difference in frequency distribution of alleles between cases and controls.

^c^ Adjusted for BMI, age at menarche, age at primiparity, number of childbirth, menopause status and family history of cancer in first-degree relatives.

^d^ Bonferroni-corrected *P* value for multiple testing.

Bold numbers denote a statistical significance at 0.05 level.

We further explored the combination effects between the aforementioned four protective polymorphisms by setting up two binary (1, 0) dummy variables. Firstly, we assessed the relative importance of the four protective tSNPs in their designated models. The adjusted OR value indicated that these four protective tSNPs affected EC susceptibility at a similar level (S4 Table). Then, individuals were categorized into five groups based on the number of protective genotypes they carried, and those without any protective genotypes were defined as the reference group. The analysis of combination effects indicated that the adjusted OR of EC for individuals carrying two protective genotypes was 0.41 (95% CI = 0.23–0.74, *P* = 0.0029). Co-existing three or four protective genotypes substantially decreased the susceptibility of EC in an almost similar degree (Table 2). Also, the protective genotypes took effect in a dose-dependent manner (*P*trend **<** 0.0001) (Table 2).

**Table 2:** Combination effects of rs1800469, rs6478974, rs10733710 in dominant model and rs4721745 in recessive model on EC susceptibility.

Number of protective genotypes^a^	Cases (%)	Controls (%)	OR (95% CI)	*P*	aOR (95% CI) ^b^	*P*^b^
Combinations of rs1800469, rs6478974, rs10733710 and rs4721745						
0 (Group 1)	49 (9.50)	32 (4.53)	Reference		Reference	
1 (Group 2)	188 (36.43)	186 (26.31)	0.66 (0.41–1.08)	0.0962	0.67 (0.36–1.22)	0.1880
2 (Group 3)	220 (42.64)	336 (47.52)	0.43 (0.27–0.69)	**0.0005**	0.41 (0.23–0.74)	**0.0029**
3 (Group 4)	54 (10.47)	137 (19.38)	0.26 (0.15–0.44)	**<0.0001**	0.24 (0.13–0.48)	**<0.0001**
4 (Group 5)	5 (0.97)	16 (2.26)	0.20 (0.07–0.61)	**0.0046**	0.24 (0.07–0.84)	**0.0255**
***P*trend<0.0001**						

EC, endometrial cancer; OR, odds ratios; CI, confidence intervals.

^a^ The genetic variants of rs1800469, rs6478974, rs10733710 and rs4721745 were considered as protective genotypes. Individuals in group 1 had no protective genotypes; in the next four groups, we pooled all individuals harboring any one protective genotype as group 2, harboring any two protective genotypes as group 3, harboring any three protective genotypes as group 4 and four protective genotypes as group 5.

^b^ Adjusted for BMI, age at menarche, age at primiparity, number of childbirth, menopause status and family history of cancer in first-degree relatives.

Bold numbers denote a statistical significance at 0.05 level.

### Association of high-order interactions among genetic variants with EC risk by CART analysis

**Fig 2:**
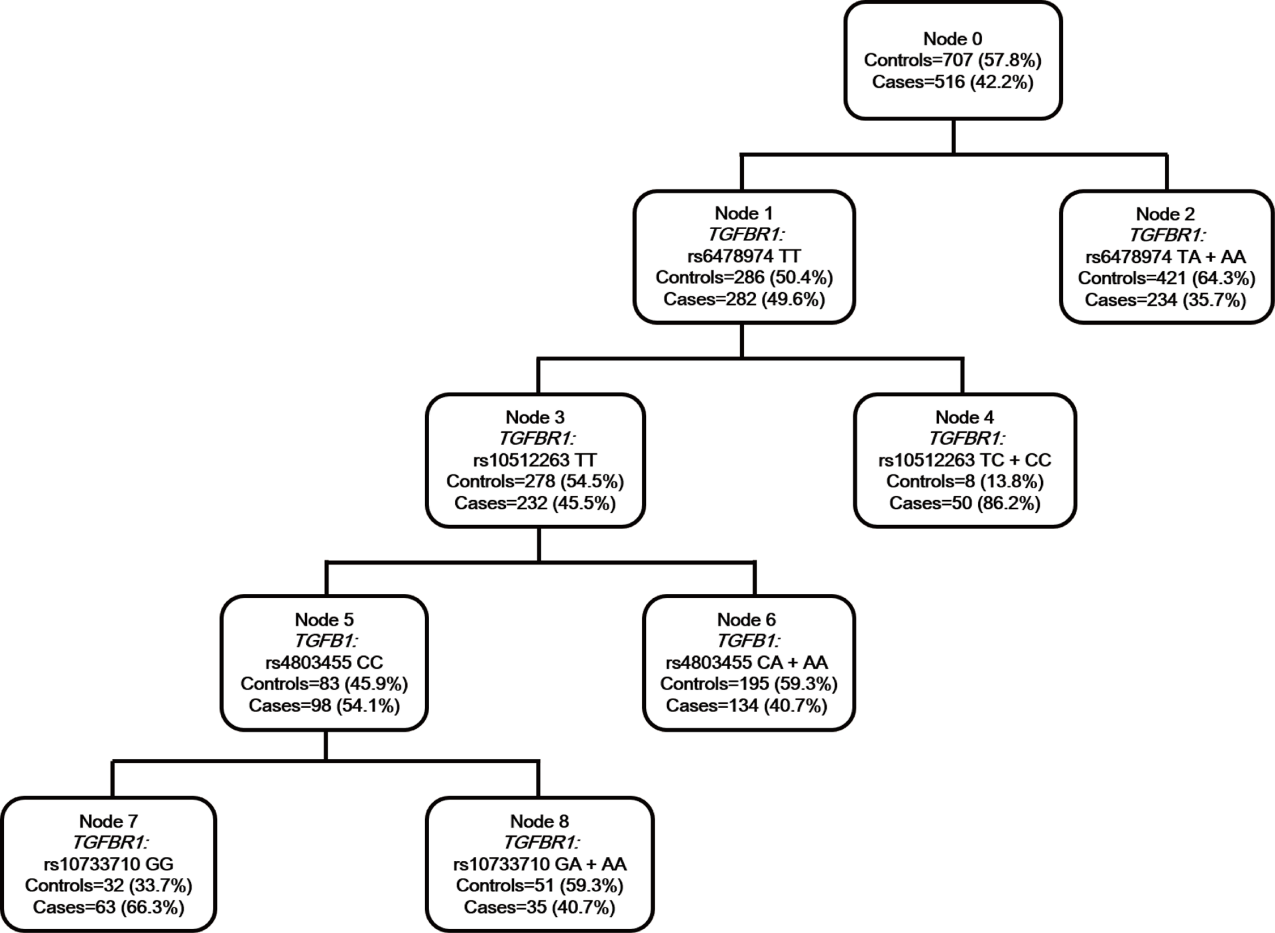
CART analysis of genetic variants in modulating EC susceptibility.

CART is a binary recursive partitioning method that produces a decision tree to identify subgroups of subjects at higher risk [18]. Fig 2 demonstrated the tree structure. The tree initiated from the total study population (node 0) and contained five terminal nodes in the final tree structure. *TGFBR1* was singled out in the first splitting node, and *TGFBR1* rs6478974 TA/AA genotype carriers had the least percentage of EC cases (35.7%), indicating that rs6478974 locus was the strongest susceptible factor for EC risk among the examined polymorphisms. Then the tree progressed along node 1 with the major allele homozygotes of SNP rs6478974. We designated node 2 as a reference node, because women in this node (with *TGFBR1* rs6478974 TA/AA genotypes) had the lowest EC risk. This tree structure revealed that individuals harboring *TGFBR1* rs6478974 TT, *TGFBR1* rs10512263 TT, *TGFB1* rs4803455 CC and *TGFBR1* rs10733710 GG genotypes (node 7) had significantly higher risk calculated by multivariate LR analysis (aOR = 3.71, 95% CI = 2.14–6.43, *P*<0.0001), and women with both the genotypes of *TGFBR1* rs6478974 TT and *TGFBR1* rs10512263 TC/CC (node 4) imparted the highest predisposition to EC risk in our population (aOR = 7.86, 95% CI = 3.42–18.07, *P*<0.0001, Table 3).

**Table 3:** Conjoint analysis of the effects of individual tSNPs on EC risk by CART.

Terminal nodes	Genotype of participants in each node	Cases (%)	Controls (%)	aOR (95% CI)^a^	*P*^a^
2	*TGFBR1* rs6478974 (TA+AA)	234 (35.7)	421 (64.3)	Reference	
6	*TGFBR1* rs6478974 (TT) +*TGFBR1* rs10512263 (TT) +	134 (40.7)	195 (59.3)	1.13 (0.81–1.59)	0.4683
	*TGFB1* rs4803455 (CA+AA)				
8	*TGFBR1* rs6478974 (TT) +*TGFBR1* rs10512263 (TT) +	35 (40.7)	51 (59.3)	1.07 (0.61–1.88)	0.8172
	*TGFB1* rs4803455 (CC) +*TGFBR1* rs10733710 (GA+AA)				
7	*TGFBR1* rs6478974 (TT) +*TGFBR1* rs10512263 (TT) +	63 (66.3)	32 (33.7)	3.71 (2.14–6.43)	**<0.0001**
	*TGFB1* rs4803455 (CC) +*TGFBR1* rs10733710 (GG)				
4	*TGFBR1* rs6478974 (TT) +*TGFBR1* rs10512263 (TC+CC)	50 (86.2)	8 (13.8)	7.86 (3.42–18.07)	**<0.0001**

tSNPs, tagging single nucleotide polymorphisms; EC, endometrial cancer; CART, classification and regression tree; OR, odds ratios; CI, confidence intervals.

^a^ Adjusted for BMI, age at menarche, age at primiparity, number of childbirth, menopause status and family history of cancer in first-degree relatives.

Bold numbers denote a statistical significance at 0.05 level.

### Association of high-order interactions among genetic variants with EC risk by MDR analysis

**Fig 3:**
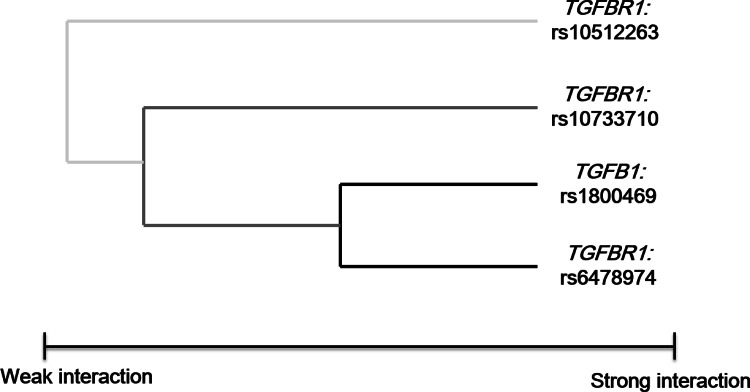
Interaction dendrogram for EC. The loci that strongly interact to each other appear close together at the branches of the tree (black line), whereas the loci with weak interaction appear distant from one another (grey line).

We applied the MDR method, a nonparametric and genetic model–free analysis, to identify interaction models. The best one-factor model generated by MDR for examining EC risk was *TGFBR1* rs6478974 (testing accuracy 0.561, CVC 9/10, Table 4), which was consistent with the first splitting node by CART analysis. The two-factor model including both *TGFB1* rs1800469 and *TGFBR1* rs6478974 was the best interaction model, which yielded the maximal CVC of 10/10 and the highest testing accuracy of 0.589. The best three-factor model including *TGFB1* rs1800469, *TGFBR1* rs6478974 and *TGFBR1* rs10733710 and the four-factor model consisting of *TGFB1* rs1800469, *TGFBR1* rs6478974, *TGFBR1* rs10512263 and *TGFBR1* rs10733710 had higher testing accuracy compared with the one-factor model (0.584, 0.575, respectively), but the CVCs were decreased (7/10, 6/10, respectively). All interaction permutation *P* value was less than 0.05. The interaction dendrogram showed that *TGFB1* rs1800469 and *TGFBR1* rs6478974 had the strongest synergistic interaction (black line), which also interacted with *TGFBR1* rs10733710 (dark grey line). Furthermore, *TGFBR1* rs10512263 had weak interaction with *TGFBR1* rs10733710, *TGFB1* rs1800469 and *TGFBR1* rs6478974 (light grey line, Fig 3). For the combined effect of *TGFB1* rs1800469 and *TGFBR1* rs6478974 in the best interaction model identified above, LR analysis demonstrated that the adjusted OR of EC was 0.43 (95% CI = 0.27–0.69, *P =* 0.0003, data not shown). The summary of these three approaches for single-locus analysis was shown in S5 Table.

**Table 4:** Association of high-order gene-gene interactions with EC risk by MDR analysis.

Number of loci	Best interaction models	Testing accuracy	Cross-validation consistency	*P* for permutation test
1	*TGFBR1* rs6478974 T>A	0.561	9/10	**<0.05**
2	*TGFB1* rs1800469 C>T,	0.589	10/10	**<0.05**
	*TGFBR1* rs6478974 T>A			
3	*TGFB1* rs1800469 C>T,	0.584	7/10	**<0.05**
	*TGFBR1* rs6478974 T>A,			
	*TGFBR1* rs10733710 G>A			
4	*TGFB1* rs1800469 C>T,	0.575	6/10	**<0.05**
	*TGFBR1* rs6478974 T>A,			
	*TGFBR1* rs10512263 T>C,			
	*TGFBR1* rs10733710 G>A			

EC, endometrial cancer; MDR, multifactor dimensionality reduction; CVC, cross-validation consistency.

Bold numbers denote a statistical significance at 0.05 level.

### Association of haplotypes and diplotypes in *TGFB1*, *TGFBR1*, *SNAI1*, *TWIST1* with EC risk by LR analysis

To further explore the modest etiological effects of polymorphisms on EC susceptibility, haplotype was reconstructed as surrogate to provide higher resolution and potentially greater statistic power [16]. In our study, *TGFB1* haplotypes (rs1800469 and rs2241716) with above 1% frequency were tested separately against the most common haplotypes, and the remaining rare haplotypes in the block (frequency < 1%) were not analyzed. The haplotype CG in block 1 was associated with increased EC risk relative to haplotype TG by univariate LR algorithm (OR = 1.60, 95% CI = 1.32–1.95, *P*˂0.0001, Table 5), and the diplotype CA-CG, carrying at-risk haplotype CG, increased about 62% of EC risk compared to the most common diplotype TG-CA (aOR = 1.62, 95% CI = 1.08–2.43, *P* = 0.0187). They did not remain significant after the Bonferroni correction. For *TGFBR1*, CACGA, harboring a protective locus rs6478974, could decrease about 42% of EC risk (aOR = 0.58, 95% CI = 0.43–0.77, *P* = 0.0003). Even after adjustment for Bonferroni-corrected multiple testing, the haplotype was still significantly associated with EC risk (Bonferroni-corrected *P***<**0.05). Furthermore, the diplotype CACGA-CTTAA, containing a protective haplotype CACGA was also associated with decreased EC risk compared with the most common diplotype TTTGG-CACGA (aOR = 0.35, 95% CI = 0.18–0.66, *P* = 0.0012), with a Bonferroni corrected *P***<**0.05 (Table 5). Haplotypes in *SNAI1* and *TWIST1* were not associated with EC susceptibility (S6 Table).

**Table 5:** Association of haplotypes and diplotypes of *TGFB1* and *TGFBR1* with EC risk.

			Cases (%)	Controls (%)	OR (95% CI)	*P*	aOR (95% CI)^a^	*P*^a^ (*P*^b^)
Block1 of *TGFB1* (rs1800469 + rs2241716)^c^								
Haplotype	TG	441 (42.73)	699 (49.43)	Reference		Reference	
	CA	306 (29.65)	457 (32.32)	0.88 (0.74–1.05)	0.1596	0.80 (0.61–1.06)	0.1249
	CG	269 (26.07)	255 (18.03)	1.60 (1.32–1.95)	**<0.0001**	1.06 (0.76–1.48)	0.7347
Diplotype	TG-CA	119 (23.06)	242 (34.23)	Reference		Reference	
	TG-TG	118 (22.87)	162 (22.91)	1.00 (0.76–1.31)	0.9851	1.15 (0.83–1.59)	0.4131
	TG-CG	78 (15.12)	130 (18.39)	0.79 (0.58–1.07)	0.1332	0.84 (0.58–1.21)	0.3440
	CA-CG	85 (16.47)	81 (11.46)	1.52 (1.10–2.12)	**0.0118**	1.62 (1.08–2.43)	**0.0187** (0.5236)
	CA-CA	51 (9.88)	67 (9.48)	1.05 (0.71–1.54)	0.8113	0.83 (0.50–1.38)	0.4815
Block of *TGFBR1* (rs10988706 + rs6478974 + rs10512263 + rs10733710 + rs334348)^c^								
Haplotype	TTTGG	363 (35.17)	573 (40.52)	Reference		Reference	
	CACGA	174 (16.86)	358 (25.32)	0.60 (0.49–0.73)	**<0.0001**	0.58 (0.43–0.77)	**0.0003 (0.0045)**
	CTTAA	147 (14.24)	252 (17.82)	0.77 (0.61–0.96)	**0.0179**	0.70 (0.48–1.02)	0.0661
	CATGA	91 (8.82)	144 (10.18)	0.85 (0.65–1.12)	0.2556	0.83 (0.54–1.27)	0.3945
	CTTGG	48 (4.65)	53 (3.75)	1.25 (0.84–1.87)	0.2701	2.01 (0.93–4.32)	0.0750
Diplotype	TTTGG-CACGA	61 (11.82)	149 (21.07)	Reference		Reference	
	TTTGG-TTTGG	77 (14.92)	117 (16.55)	0.88 (0.65–1.21)	0.4422	0.82 (0.55–1.22)	0.3336
	TTTGG-CTTAA	59 (11.43)	103 (14.57)	0.76 (0.54–1.07)	0.1111	0.51 (0.32–0.81)	**0.0045** (0.1260)
	CACGA-CTTAA	19 (3.68)	70 (9.90)	0.35 (0.21–0.59)	**<0.0001**	0.35 (0.18–0.66)	**0.0012 (0.0336)**
	TTTGG-CATGA	29 (5.62)	53 (7.50)	0.74 (0.46–1.17)	0.1966	0.78 (0.45–1.36)	0.3839
	CACGA-CACGA	25 (4.84)	40 (5.66)	0.85 (0.51–1.42)	0.5319	0.83 (0.45–1.55)	0.5655
	CACGA-CATGA	24 (4.65)	37 (5.23)	0.88 (0.52–1.50)	0.6443	1.11 (0.59–2.09)	0.7550
	CTTAA-CTTAA	14 (2.71)	22 (3.11)	0.87 (0.44–1.71)	0.6840	1.07 (0.50–2.32)	0.8550

EC, endometrial cancer; OR, odds ratios; CI, confidence intervals.

^a^ Adjusted for BMI, age at menarche, age at primiparity, number of childbirth, menopause status and family history of cancer in first-degree relatives.

^b^ Bonferroni-corrected *P* value for multiple testing.

^c^ Haplotypes and diplotypes with frequency less than 1% were omitted.

Bold numbers denote a statistical significance at 0.05 level.

### Association of interactions among genetic variants and environmental factors with EC risk

Given that long-term exposure to estrogen, cancer history in first-degree relatives and overweight are clinical EC risk factors [19], we conducted analysis in stratified population to explore whether the associations of genetic variants with EC risk were modified by these clinical risk factors. Table 6 showed that women harboring *TGFBR1* protective haplotype CACGA had an even lower EC risk among those with longer menarche-FFTP intervals (˃11 years [20], aOR = 0.49, 95% CI = 0.31–0.75, *P* = 0.0012) and without family history of cancer (aOR = 0.49, 95% CI = 0.30–0.80, *P* = 0.0044). Also, CACGA carriers had a bit lower EC risk in BMI˂24 subgroup than in BMI≥24 subgroup (BMI˂24, aOR = 0.62, 95% CI = 0.40–0.96, *P* = 0.0323; BMI≥24, aOR = 0.70, 95% CI = 0.51–0.96, *P* = 0.0255). Moreover, carriers of this haplotype showed the lowest EC risk among women with longer menarche-FFTP intervals and BMI˂24 (aOR = 0.39, 95% CI = 0.17–0.90, *P* = 0.0275) (Table 6).

**Table 6:** Stratified analysis between protective haplotype CACGA of *TGFBR1* and EC risk by family history of cancer, BMI status and menarche-FFTP intervals.

	All			Menarche-FFTP intervals^a^ ≤ 11 years			Menarche-FFTP intervals^a^ ˃ 11 years		
	Cases (%)/Controls (%)	aOR (95% CI)^b^	*P*^b^	Cases (%)/Controls (%)	aOR (95% CI)^b^	*P*^b^	Cases (%)/Controls (%)	aOR (95% CI)^b^	*P*^b^
All									
Other haplotypes	858 (83.14)/1056 (74.68)	Reference		477 (81.12)/695 (75.87)	Reference		381 (85.81)/361 (72.49)	Reference	
CACGA	174 (16.86)/358 (25.32)	**0.66 (0.51–0.84)**	**0.0008**	111 (18.88)/221 (24.13)	0.79 (0.58–1.08)	0.1330	63 (14.19)/137 (27.51)	**0.49 (0.31–0.75)**	**0.0012**
Family history of cancer (yes)									
Other haplotypes	131 (79.88)/216 (75.00)	Reference		59 (73.75)/107 (75.35)	Reference		72 (85.71)/109 (74.66)	Reference	
CACGA	33 (20.12)/72 (25.00)	0.88 (0.49–1.56)	0.6573	21 (26.25)/35 (24.65)	1.28 (0.58–2.84)	0.5373	12 (14.29)/37 (25.34)	0.70 (0.25–1.98)	0.5065
Family history of cancer (no)									
Other haplotypes	727 (83.76)/840 (74.60)	Reference		418 (82.28)/588 (75.97)	Reference		309 (85.83)/252 (71.59)	Reference	
CACGA	141 (16.24)/286 (25.40)	**0.61 (0.46–0.81)**	**0.0005**	90 (17.72)/186 (24.03)	0.73 (0.52–1.02)	0.0662	51 (14.17)/100 (28.41)	**0.49 (0.30–0.80)**	**0.0044**
BMI ˂24									
Other haplotypes	286 (83.14)/409 (74.91)	Reference		179 (81.36)/252 (77.78)	Reference		107 (86.29)/157 (70.72)	Reference	
CACGA	58 (16.86)/137 (25.09)	**0.62 (0.40–0.96)**	**0.0323**	41 (18.64)/72 (22.22)	0.75 (0.43–1.31)	0.3075	17 (13.71)/65 (29.28)	**0.39 (0.17–0.90)**	**0.0275**
BMI ≥24									
Other haplotypes	572 (83.14)/647 (74.54)	Reference		298 (80.98)/443 (74.83)	Reference		274 (85.63)/204 (73.91)	Reference	
CACGA	116 (16.86)/221 (25.46)	**0.70 (0.51–0.96)**	**0.0255**	70 (19.02)/149 (25.17)	0.79 (0.54–1.15)	0.2227	46 (14.38)/72 (26.09)	**0.54 (0.32–0.93)**	**0.0269**

EC, endometrial cancer; BMI, body mass index; FFTP, first full term pregnancy; OR, odds ratios; CI, confidence intervals.

^a^ Both parous and nulliparous women were included. This parameter was measured by age at FFTP minus age at menarche in all parous women, age at menopause minus age at menarche in postmenopausal nulliparous women, and age at EC diagnosed (case) or age at interview (control) minus age at menarche in premenopausal nulliparous women.

^b^ Adjusted for BMI, age at menarche, age at primiparity, number of childbirth, menopause status and family history of cancer in first-degree relatives.

Bold numbers denote a statistical significance at 0.05 level.

## Discussion

In this study, we applied multiple strategies including LR, CART and MDR approaches to systematically evaluate the association of EC susceptibility with germline variants in TGF-β signaling related genes *TGFB1*, *TGFBR1*, *SNAI1* and *TWIST1* among Chinese Han women.

In single-locus analysis using multivariate LR, five polymorphisms, rs1800469 in *TGFB1*, rs6478974 and rs10733710 in *TGFBR1*, rs4721746 and rs4721745 in *TWIST1* showed significant association with EC susceptibility. Although LR has been widely used in multivariate gene-gene or gene-environment interactions, it cannot fully characterize them because of the sparseness of data in high dimensions. Moreover, its statistic power would decrease and type II errors would increase in relatively small sample size [21]. So the non-parametric CART and MDR analysis were employed in high-order gene-gene interactions to examine particular combination effects of genetic variants. In this study, CART analysis indicated that the most important splitting variable was *TGFBR1* rs6478974, followed by *TGFBR1* rs10512263. The MDR method, which reduces the genotype parameters from multi- dimension to one dimension, demonstrated that *TGFB1* rs1800469 and *TGFBR1* rs6478974 together were the best interactional polymorphisms to examine EC risk.

All the three approaches in single-locus analysis consistently indicated that the genotype *TGFBR1* rs6478974 TA/AA (located in intron 1) had the strongest protective effect on EC susceptibility. Until now, common variants were seldom reported in the exons or functional regions of *TGFBR1* to have clear functional relevance. Although the vast majority of SNPs are located in the genomic non-coding regions, new evidence suggests that SNPs, located in gene promoter or regulatory regions, play critical roles in regulating the nature and timing of gene expression [22]. Chen J *et al* found that rs6478974 was associated with increased risk of gastric cancer in Chinese population (in dominant model: aOR = 1.36, 95% CI = 1.14–1.63; in additive model: aOR = 1.23, 95% CI = 1.08–1.40) [23]. They discovered that rs6478974 was in moderate LD with rs334348 and rs1590 (in the 3’-UTR, both r^2^ = 0.504) using online software SNPinfo (http://manticore.niehs.nih.gov/cgi-bin/snpinfo/snpfunc.cgi), and these two loci probably regulated miRNAs binding and influenced gastric cancer development. Because TβRI inhibits cell growth during early tumorigenesis [9, 24], we speculate that individuals carrying *TGFBR1* rs6478974 TA/AA express higher levels of TβRI than TT genotype carriers, and therefore have lower susceptibility to EC. We also found that individuals with both the genotypes of *TGFBR1* rs6478974 TT and *TGFBR1* rs10512263 TC/CC had higher susceptibility compared with those harboring the genotype *TGFBR1* rs6478974 TA/AA by CART analysis, which indicates that rs10512263 could be a risk locus. A two-stage case-control study of gastric cancer (the first stage of cases/controls = 650/683; the second stage of cases/controls = 484/348) showed that rs10512263 in dominant models (CT/CC vs. TT) was significantly associated with increased risk of gastric cancer in Chinese population [23], which is consistent with our results. But Scollen S *et al* discovered that the minor allele C of rs10512263 had a protective effect on breast cancer susceptibility (OR = 0.87, 95% CI = 0.81–0.95, *P* = 0.001) in meta-analysis of the SEARCH and PBCS studies [25]. The discrepancies among these results could be due to the ethnic diversity of populations and complicated environmental factors.

In *TGFB1*, we observed that the T allele of rs1800469 (C˃T at the 5’UTR region) was associated with decreased EC susceptibility under dominant model, which was consistent with the result in gastric cancer among the same ethnic population (cases/controls = 675/704, aOR = 0.65, 95% CI = 0.52–0.82) [26]. Our MDR analysis demonstrated that the combined genetic variants of *TGFB1* rs1800469 and *TGFBR1* rs6478974, the best interaction model, decreased the EC risk, which was in accordance with the results analyzed by LR. It was reported that the T allele of rs1800469 could enhance the affinity of its promoter with some transcriptional factors such as Yin Yang 1 (YY1), and increase the expression of TGF-β1 [27, 28]. Moreover, Grainger DJ *et al* showed that the concentration of TGF-β1 in plasma was extremely higher in T allele carriers than C allele carriers among UK population [29]. The polymorphism *TGFB1* rs1800469 may perform its protective function during early tumorigenesis by altering the expression of TGF-β1.

In *TWIST1*, we discovered that the variant genotypes of rs4721746 and rs4721745, both locating in the 3’ flanking regions, had opposite effects on EC risk in our population. When using web-based functional annotation tool F-SNP (http://compbio.cs.queensu.ca/F-SNP/) [30], these two polymorphisms were both predicted to influence transcriptional regulation by TFSearch and Consite tools (functional significance scores = 0.239; 0.208, respectively). Further studies in other population are needed to verify our findings.

Haplotype-based approach may have greater power than single-locus analysis when SNPs are in strong LD and would provide additional statistical power to detect genes involved in complex trait diseases [31, 32]. In our haplotype-reconstruction association study, we found that *TGFB1* haplotype CG and diplotype CA-CG were both associated with increased EC susceptibility. In *TGFBR1*, haplotype CACGA and diplotype CACGA-CTTAA decreased EC risk. Also, we observed significant joint effects of haplotype CACGA, family history of cancer, BMI status and estrogen exposure in stratified analysis. Haplotype CACGA, harboring a protective allele A of rs6478974, decreased the risk of EC regardless of what the environmental factors were, which further indicated that A allele of rs6478974 might be the most important protective locus in our population. If these haplotypes and diplotypes could be proved in other populations, they can be used as molecular makers for the estimation of EC risk, and can also provide some clues for finding causal SNPs.

There are three main strengths in our study. First, in single-locus analysis, we not only used traditional LR, but also CART and MDR approaches to identify high-order interactions while overcoming LR’s shortcomings, such as inaccurate parameter estimates and low power for detecting interactions. Second, we performed gene-wide analysis of tSNPs that covered all common SNPs of the four genes. Third, we reconstructed haplotype blocks according to our genotyping data in controls, which could guarantee the reasonable division of haplotype blocks. However, our study has several limitations. First, our sample size was relatively small, and the number of individuals was even smaller when the data were stratified. Second, the causal genetic variants hidden behind the association have not been revealed, and a further fine mapping study with high-density SNPs within the target region would be helpful in identifying the causal variants.

## Supporting Information

S1 Table Characteristics of EC patients and controls. (DOC)

S2 Table Hardy-Weinberg equilibrium of the 19 tSNPs in *TGFB1*, *TGFBR1*, *SNAI1*, *TWIST1*. (DOC)

S3 Table Univariate and multivariate analysis of the association of candidate tSNPs with EC risk. (DOC)

S4 Table Risk of EC associated with the combination of four protective tSNPs by multivariate analysis. (DOC)

S5 Table Comparative results of multivariate LR, CART and MDR analysis in single-locus SNPs. (DOC)

S6 Table Association of haplotypes and diplotypes of *SNAI1* and *TWIST1* with EC risk. (DOC)
