# Supplementary material for: Genetic Polymorphisms of TGFB1, TGFBR1, SNAI1 and TWIST1 Are Associated with Endometrial Cancer Susceptibility in Chinese Han Women
Source: PLoS One. 2016 May 12;11(5):e0155270. doi: 10.1371/journal.pone.0155270 (PMC4865208; doi:10.1371/journal.pone.0155270)
Supplement: S1 Table — (DOC) [file pone.0155270.s001.doc]

**Table S1.** Characteristics of EC patients and controls.

| Variables | Cases, n=516 | Controls, n=707 | *P* |
| --- | --- | --- | --- |
| Age, years (mean±SD) | 56.09±10.47 | 55.94±6.56 | 0.7528 |
| Age, n (%) |  |  | 0.1263 |
| ˂55 | 229 (44.38) | 345 (48.80) |  |
| ≥55 | 287 (55.62) | 362 (51.20) |  |
| BMI, (mean±SD) | 25.85±4.11 | 24.92±3.17 | **<0.0001** |
| Age at menarche, years (mean±SD) | 14.76±1.84 | 15.58±1.94 | **<0.0001** |
| Menarche status, n (%) |  |  | **<0.0001** |
| ˂15 | 255 (49.42) | 224 (31.68) |  |
| ≥15 | 261 (50.58) | 483 (68.32) |  |
| Age at menopause, years (mean±SD) | 50.51±3.52 | 49.61±3.53 | **0.0002** |
| Menopause status, n (%) |  |  | **<0.0001** |
| Premenopause | 185 (35.85) | 122 (17.26) |  |
| Postmenopause | 331 (64.15) | 585 (82.74) |  |
| Age at first full-term pregnancy (FFTP), years (mean±SD) | 25.17±3.36 | 25.54±2.94 | **0.0451** |
| [Number](http://www.iciba.com/number/) [of](http://www.iciba.com/of/) child birth, n (%) |  |  | **˂0.0001** |
| 0 | 60 (11.63) | 8 (1.13) |  |
| 1 | 186 (36.05) | 365 (51.63) |  |
| ≥2 | 270 (52.33) | 334 (47.24) |  |
| Family history of cancer in first-degree relatives, n (%) |  |  | **0.0464** |
| Yes | 82 (15.89) | 144 (20.37) |  |
| No | 434 (84.11) | 563 (79.63) |  |
| Smoking history, n (%) |  |  | 0.6212 |
| Yes | 19 (3.68) | 30 (4.24) |  |
| No | 497 (96.32) | 677 (95.76) |  |

EC, endometrial cancer; BMI, body mass index; FFTP, first full term pregnancy.

Bold numbers denote a statistical significance at 0.05 level.
