# Supplementary material for: Genetic Polymorphisms of TGFB1, TGFBR1, SNAI1 and TWIST1 Are Associated with Endometrial Cancer Susceptibility in Chinese Han Women
Source: PLoS One. 2016 May 12;11(5):e0155270. doi: 10.1371/journal.pone.0155270 (PMC4865208; doi:10.1371/journal.pone.0155270)
Supplement: S2 Table — (DOC) [file pone.0155270.s002.doc]

***Table S2.*** *Hardy-Weinberg equilibrium of the 19 tSNPs in TGFB1, TGFBR1, SNAI1, TWIST1.*

|  | SNPs | *P* | HWE |
| --- | --- | --- | --- |
| *TGFB1* | rs1800469 | 0.1734 | Yes |
|  | rs2241716 | 0.1962 | Yes |
|  | rs4803455 | 0.7611 | Yes |
|  | rs747857 | 1.0000 | Yes |
|  | rs12983047 | 0.9358 | Yes |
|  | rs10417924 | 0.8743 | Yes |
|  | rs12981053 | 0.3951 | Yes |
| *TGFBR1* | rs10988706 | 0.8793 | Yes |
|  | rs6478974 | 0.8695 | Yes |
|  | rs10512263 | 0.3751 | Yes |
|  | rs10733710 | 1.0000 | Yes |
|  | rs334348 | 0.8758 | Yes |
| *SNAI1* | rs6125849 | 0.6997 | Yes |
|  | rs4647959 | 0.6357 | Yes |
|  | rs6020178 | 0.1324 | Yes |
| *TWIST1* | rs2285682 | 0.7361 | Yes |
|  | rs2285681 | 0.7680 | Yes |
|  | rs4721746 | 0.3025 | Yes |
|  | rs4721745 | 0.8750 | Yes |

tSNPs, tagging single nucleotide polymorphisms; HWE, Hardy-Weinberg equilibrium.
