# Supplementary material for: Genetic Polymorphisms of TGFB1, TGFBR1, SNAI1 and TWIST1 Are Associated with Endometrial Cancer Susceptibility in Chinese Han Women
Source: PLoS One. 2016 May 12;11(5):e0155270. doi: 10.1371/journal.pone.0155270 (PMC4865208; doi:10.1371/journal.pone.0155270)
Supplement: S3 Table — (DOC) [file pone.0155270.s003.doc]

**Table S3.** Univariate and multivariate analysis of the association of candidate tSNPs with EC risk.

| Gene | SNPs | Genotype | Cases (%) | | Controls (%) | | *P*^a^ | *P*^b^ | *P*_trend_ | OR (95% CI) | *P* | aOR (95% CI)^c^ | *P*^c^ |
| --- | --- | --- | --- | --- | --- | --- | --- | --- | --- | --- | --- | --- | --- |
| *TGFB1* | rs2241716 | GG | 249 (48.26) | | 314 (44.41) | | 0.2136 |  | 0.4835 | Reference |  | Reference |  |
|  |  | GA | 212 (41.09) | | 326 (46.11) | |  |  |  | 0.82 (0.65-1.04) | 0.1051 | 0.87 (0.65-1.16) | 0.3406 |
|  |  | AA | 55 (10.66) | | 67 (9.48) | |  |  |  | 1.04 (0.70-1.53) | 0.8632 | 0.80 (0.75-0.87) | 0.6656 |
|  |  | A allele frequency | 322 (31.20) | | 460 (32.53) | |  | 0.4860 |  |  |  |  |  |
|  |  | GA/AA vs. GG (dominant model) | | |  | |  |  |  | 0.86 (0.68-1.08) | 0.1831 | 0.87 (0.66-1.15) | 0.3358 |
|  |  | AA vs. GG/GA (recessive model) | | |  | |  |  |  | 1.14 (0.78-1.66) | 0.4959 | 0.91 (0.56-1.49) | 0.7080 |
| *TGFB1* | rs4803455 | CC | 207 (40.12) | | 242 (34.23) | | 0.0626 |  | 0.1918 | Reference |  | Reference |  |
|  |  | CA | 220 (42.64) | | 347 (49.08) | |  |  |  | 0.74 (0.58-0.95) | **0.0193** | 0.76 (0.56-1.03) | 0.0805 |
|  |  | AA | 89 (17.25) | | 118 (16.69) | |  |  |  | 0.88 (0.63-1.23) | 0.4574 | 0.88 (0.58-1.33) | 0.5426 |
|  |  | A allele frequency | 398 (38.57) | | 583 (41.23) | |  | 0.1842 |  |  |  |  |  |
|  |  | CA/AA vs. CC (dominant model) | | |  | |  |  |  | 0.78 (0.61-0.98) | **0.0351** | 0.79 (0.59-1.05) | 0.1088 |
|  |  | AA vs. CC/CA (recessive model) | | |  | |  |  |  | 1.04 (0.77-1.41) | 0.7969 | 0.98 (0.67-1.43) | 0.9092 |
| *TGFB1* | rs747857 | CC | 451 (87.40) | | 618 (87.41) | | 0.9261 |  | 0.9352 | Reference |  | Reference |  |
|  |  | CT | 62 (12.02) | | 86 (12.16) | |  |  |  | 0.99 (0.70-1.40) | 0.9453 | 1.13 (0.74-1.72) | 0.5650 |
|  |  | TT | 3 (0.58) | | 3 (0.42) | |  |  |  | 1.37 (0.28-6.82) | 0.7004 | 0.68 (0.06-8.23) | 0.7611 |
|  |  | T allele frequency | 68 (6.59) | | 92 (6.51) | |  | 0.9348 |  |  |  |  |  |
|  |  | CT/TT vs. CC (dominant model) | | |  | |  |  |  | 1.00 (0.71-1.41) | 0.9965 | 1.12 (0.74-1.69) | 0.6030 |
|  |  | TT vs. CC/CT (recessive model) | | |  | |  |  |  | 1.37 (0.28-6.83) | 0.6990 | 0.67 (0.06-8.10) | 0.7520 |
| *TGFB1* | rs12983047 | AA | 217 (42.05) | | 296 (41.87) | | 0.5300 |  | 0.6409 | Reference |  | Reference |  |
|  |  | AG | 225 (43.60) | | 324 (45.83) | |  |  |  | 0.95 (0.74-1.21) | 0.6636 | 0.96 (0.71-1.29) | 0.7681 |
|  |  | GG | 74 (14.34) | | 87 (12.31) | |  |  |  | 1.16 (0.81-1.66) | 0.4132 | 1.16 (0.75-1.79) | 0.4983 |
|  |  | G allele frequency | 373 (36.14) | | 498 (35.22) | |  | 0.6373 |  |  |  |  |  |
|  |  | AG/GG vs. AA (dominant model) | | |  | |  |  |  | 0.99 (0.79-1.25) | 0.9478 | 0.99 (0.75-1.32) | 0.9534 |
|  |  | GG vs. AA/AG (recessive model) | | |  | |  |  |  | 1.19 (0.86-1.67) | 0.2988 | 1.19 (0.79-1.78) | 0.4025 |
| *TGFB1* | rs10417924 | CC | 372 (72.09) | | 530 (74.96) | | 0.5015 |  | 0.2401 | Reference |  | Reference |  |
|  |  | CT | 132 (25.58) | | 164 (23.20) | |  |  |  | 1.15 (0.88-1.49) | 0.3108 | 1.18 (0.85-1.64) | 0.3102 |
|  |  | TT | 12 (2.33) | | 13 (1.84) | |  |  |  | 1.32 (0.59-2.91) | 0.4998 | 1.16 (0.46-2.91) | 0.7605 |
|  |  | T allele frequency | 156 (15.12) | | 190 (13.44) | |  | 0.2392 |  |  |  |  |  |
|  |  | CT/TT vs. CC (dominant model) | | |  | |  |  |  | 1.16 (0.90-1.50) | 0.2598 | 1.18 (0.86-1.62) | 0.3000 |
|  |  | TT vs. CC/CT (recessive model) | | |  | |  |  |  | 1.27 (0.58-2.81) | 0.5533 | 1.09 (0.43-2.77) | 0.8524 |
| *TGFB1* | rs12981053 | CC | 276 (53.49) | | 372 (52.62) | | 0.2676 |  | 0.6957 | Reference |  | Reference |  |
|  |  | CT | 184 (35.66) | | 275 (38.90) | |  |  |  | 0.90 (0.71-1.15) | 0.4047 | 1.02 (0.74-1.40) | 0.9291 |
|  |  | TT | 56 (10.85) | | 60 (8.49) | |  |  |  | 1.26 (0.85-1.87) | 0.2561 | 0.82 (0.45-1.49) | 0.5085 |
|  |  | T allele frequency | 296 (28.68) | | 395 (27.93) | |  | 0.6852 |  |  |  |  |  |
|  |  | TT/CT vs. CC (dominant model) |  | |  | |  |  |  | 0.88 (0.66-1. 19) | 0.4139 | 0.95 (0.72-1.26) | 0.7337 |
|  |  | TT vs. CC/CT (recessive model) |  | |  | |  |  |  | 1.27 (0.79-2.06) | 0.3263 | 1.34 (0.84-2.13) | 0.2182 |
| *TGFBR1* | rs10988706 | CC | 181 (35.08) | | 249 (35.22) | | 0.1564 |  | 0.3050 | Reference |  | Reference |  |
|  |  | CT | 227 (43.99) | | 339 (47.95) | |  |  |  | 0.92 (0.71-1.19) | 0.5277 | 0.97 (0.71-1.33) | 0.8387 |
|  |  | TT | 108 (20.93) | | 119 (16.83) | |  |  |  | 1.25 (0.90-1.73) | 0.1784 | 1.23 (0.83-1.84) | 0.3020 |
|  |  | T allele frequency | 443 (42.93) | | 577 (40.81) | |  | 0.2936 |  |  |  |  |  |
|  |  | CT/TT vs. CC (dominant model) | | |  | |  |  |  | 1.01 (0.79-1.28) | 0.9591 | 1.09 (0.81-1.46) | 0.5885 |
|  |  | TT vs. CC/CT (recessive model) | | |  | |  |  |  | 1.31 (0.98-1.75) | 0.0691 | 1.26 (0.88-1.80) | 0.2101 |
| *TGFBR1* | rs334348 | AA | 161 (31.20) | | 214 (30.27) | | 0.3736 |  | 0.6632 | Reference |  | Reference |  |
|  |  | AG | 238 (46.12) | | 352 (49.79) | |  |  |  | 0.90 (0.69-1.17) | 0.4251 | 0.94 (0.68-1.29) | 0.6846 |
|  |  | GG | 117 (22.67) | | 141 (19.94) | |  |  |  | 1.10 (0.80-1.52) | 0.5474 | 1.15 (0.78-1.70) | 0.4829 |
|  |  | G allele frequency | 472 (45.74) | | 634 (44.84) | |  | 0.6590 |  |  |  |  |  |
|  |  | AG/GG vs. AA (dominant model) | | |  | |  |  |  | 0.96 (0.75-1.22) | 0.7265 | 1.00 (0.74-1.35) | 0.9895 |
|  |  | GG vs. AA/AG (recessive model) | | |  | |  |  |  | 1.18 (0.85-1.68) | 0.2479 | 1.20 (0.79-1.78) | 0.2992 |
| *SNAI1* | rs6125849 | GG | | 145 (28.10) | | 198 (28.01) | 0.8371 |  | 0.7645 | Reference |  | Reference |  |
|  |  | GA | | 254 (49.22) | | 358 (50.64) |  |  |  | 0.97 (0.74-1.27) | 0.8171 | 0.96 (0.69-1.34) | 0.8040 |
|  |  | AA | | 117 (22.67) | | 151 (21.36) |  |  |  | 1.06 (0.77-1.46) | 0.7307 | 1.08 (0.72-1.61) | 0.7175 |
|  |  | A allele frequency | | 488 (47.29) | | 660 (46.68) |  | 0.7650 |  |  |  |  |  |
|  |  | GA/AA vs. GG (dominant model) | | | |  |  |  |  | 1.00 (0.77-1.28) | 0.9708 | 0.99 (0.72-1.37) | 0.9684 |
|  |  | AA vs. GG/GA (recessive model) | | | |  |  |  |  | 1.08 (0.82-1.42) | 0.5814 | 1.11 (0.79-1.55) | 0.5511 |
| *SNAI1* | rs4647959 | TT | | 434 (84.11) | | 590 (83.45) | 0.6048 |  | 0.9493 | Reference |  | Reference |  |
|  |  | TC | | 75 (14.53) | | 111 (15.70) |  |  |  | 0.92 (0.67-1.26) | 0.6006 | 0.80 (0.53-1.20) | 0.2716 |
|  |  | CC | | 7 (1.36) | | 6 (0.85) |  |  |  | 1.59 (0.53-4.75) | 0.4101 | 3.16 (0.88-11.27) | 0.0766 |
|  |  | C allele frequency | | 89 (8.62) | | 123 (8.70) |  | 0.9483 |  |  |  |  |  |
|  |  | TC/CC vs. TT (dominant model) | | | |  |  |  |  | 0.95 (0.70-1.30) | 0.7590 | 0.93 (0.63-1.37) | 0.7245 |
|  |  | CC vs. TT/TC (recessive model) | | | |  |  |  |  | 1.61 (0.54-4.81) | 0.3966 | 3.40 (0.95-12.20) | 0.0600 |
| *SNAI1* | rs6020178 | TT | | 362 (70.16) | | 521 (73.69) | 0.3462 |  | 0.2536 | Reference |  | Reference |  |
|  |  | TC | | 140 (27.13) | | 166 (23.48) |  |  |  | 1.21 (0.93-1.58) | 0.1470 | 1.05 (0.76-1.45) | 0.7591 |
|  |  | CC | | 14 (2.71) | | 20 (2.83) |  |  |  | 1.01 (0.50-2.02) | 0.9833 | 1.34 (0.60-3.00) | 0.4829 |
|  |  | C allele frequency | | 168 (16.28) | | 206 (14.57) |  | 0.2457 |  |  |  |  |  |
|  |  | TC/CC vs. TT (dominant model) | | | |  |  |  |  | 1.19 (0.93-1.53) | 0.1730 | 1. 08 (0.79-1.48) | 0.6216 |
|  |  | CC vs. TT/TC (recessive model) | | | |  |  |  |  | 0.96 (0.48-1.92) | 0.9036 | 1.32 (0.59-2.95) | 0.5006 |
| *TWIST1* | rs2285682 | TT | | 390 (75.58) | | 537 (75.95) | 0.4915 |  | 0.6425 | Reference |  | Reference |  |
|  |  | TG | | 114 (22.09) | | 160 (22.63) |  |  |  | 0.98 (0.75-1.29) | 0.8909 | 0.98 (0.71-1.37) | 0.9259 |
|  |  | GG | | 12 (2.33) | | 10 (1.41) |  |  |  | 1.65 (0.71-3.86) | 0.2465 | 1.83 (0.66-5.06) | 0.2424 |
|  |  | G allele frequency | | 138 (13.37) | | 180 (12.73) |  | 0.6409 |  |  |  |  |  |
|  |  | TG/GG vs. TT (dominant model) | | | |  |  |  |  | 1.02 (0.78-1.33) | 0.8802 | 1.05 (0.76-1.45) | 0.7723 |
|  |  | GG vs.TT/TG (recessive model) | | | |  |  |  |  | 1.66 (0.71-3.87) | 0.2412 | 1.84 (0.67-5.06) | 0.2381 |
| *TWIST1* | rs2285681 | GG | | 264 (51.16) | | 374 (52.90) | 0.8298 |  | 0.6117 | Reference |  | Reference |  |
|  |  | GC | | 215 (41.67) | | 283 (40.03) |  |  |  | 1.08 (0.85-1.36) | 0.5435 | 1.09 (0.81-1.46) | 0.5617 |
|  |  | CC | | 37 (7.17) | | 50 (7.07) |  |  |  | 1.05 (0.67-1.65) | 0.8385 | 0.84 (0.48-1.47) | 0.5441 |
|  |  | C allele frequency | | 289 (28.00) | | 383 (27.09) |  | 0.6156 |  |  |  |  |  |
|  |  | GC/CC vs. GG (dominant model) | | | |  |  |  |  | 1.07 (0.85-1.35) | 0.5481 | 1.05 (0.79-1.39) | 0.7429 |
|  |  | CC vs. GG/GC (recessive model) | | | |  |  |  |  | 1.02 (0.65-1.58) | 0.9472 | 0.81 (0.47-1.40) | 0.4461 |

tSNPs, tagging single nucleotide polymorphisms; EC, endometrial cancer; OR, odds ratios; CI, confidence intervals.

^a^ Two-sided χ2 test for difference in frequency distribution of genotypes between cases and controls.

^b^ Two-sided χ2 test for difference in frequency distribution of alleles between cases and controls.

^c^ Adjusted for BMI, age at menarche, age at primiparity, [number](http://www.iciba.com/number/) [of](http://www.iciba.com/of/) childbirth, menopause status and family history of cancer in first-degree relatives.

Bold numbers denote a statistical significance at 0.05 level.
