# Supplementary material for: Genetic Polymorphisms of TGFB1, TGFBR1, SNAI1 and TWIST1 Are Associated with Endometrial Cancer Susceptibility in Chinese Han Women
Source: PLoS One. 2016 May 12;11(5):e0155270. doi: 10.1371/journal.pone.0155270 (PMC4865208; doi:10.1371/journal.pone.0155270)
Supplement: S4 Table — (DOC) [file pone.0155270.s004.doc]

**Table S4.** Risk of EC associated with the combination of four protective tSNPs by multivariate analysis.

| Gene | SNP | OR (95% CI) | *P* | aOR (95% CI)^a^ | *P*^a^ |
| --- | --- | --- | --- | --- | --- |
| *TGFB1* | rs1800469 (dominant model) | 0.54 (0.42-0.70) | **<0.0001** | 0.68 (0.50-0.94) | **0.0180** |
| *TGFBR1* | rs6478974 (dominant model) | 0.52 (0.41-0.66) | **<0.0001** | 0.57 (0.43-0.77) | **0.0002** |
| *TGFBR1* | rs10733710 (dominant model) | 0.75 (0.58-0.97) | **0.0277** | 0.61 (0.44-0.84) | **0.0025** |
| *TWIST1* | rs4721745 (recessive model) | 0.90 (0.66-1.21) | 0.4678 | 0.67 (0.46-0.98) | **0.0378** |

tSNPs, tagging single nucleotide polymorphisms; EC, endometrial cancer; OR, odds ratios; CI, confidence intervals.

^a^ Adjusted for BMI, age at menarche, age at primiparity, [number](http://www.iciba.com/number/) [of](http://www.iciba.com/of/) childbirth, menopause status and family history of cancer in first-degree relatives.

Bold numbers denote a statistical significance at 0.05 level.
