# Supplementary material for: Genetic Polymorphisms of TGFB1, TGFBR1, SNAI1 and TWIST1 Are Associated with Endometrial Cancer Susceptibility in Chinese Han Women
Source: PLoS One. 2016 May 12;11(5):e0155270. doi: 10.1371/journal.pone.0155270 (PMC4865208; doi:10.1371/journal.pone.0155270)
Supplement: S5 Table — (DOC) [file pone.0155270.s005.doc]

**Table S5**. Comparative results of multivariate LR, CART and MDR analysis in single-locus SNPs.

| Polymorphisms | Multivariate LR | CART | MDR |
| --- | --- | --- | --- |
| *TGFB1* rs1800469 | Significant protective | - | Two-, three- and four-factor models |
| *TGFB1* rs4803455 | - | 3^rd^ splitting node | - |
| *TGFBR1* rs6478974 | Significant protective | 1^st^ splitting node | One-, two-, three- and four-factor models |
| *TGFBR1* rs10512263 | - | 2^nd^ splitting node | Four-factor model |
| *TGFBR1* rs10733710 | Significant protective | 4^th^ splitting node | Three- and four-factor models |
| *TWIST1* rs4721746 | Significant risk | - | - |
| *TWIST1* rs4721745 | Significant protective |  | - |

tSNPs, tagging single nucleotide polymorphisms; LR, logistic regression; CART, classification and regression tree; MDR, multifactor dimensionality reduction.
