# Supplementary material for: Genetic Polymorphisms of TGFB1, TGFBR1, SNAI1 and TWIST1 Are Associated with Endometrial Cancer Susceptibility in Chinese Han Women
Source: PLoS One. 2016 May 12;11(5):e0155270. doi: 10.1371/journal.pone.0155270 (PMC4865208; doi:10.1371/journal.pone.0155270)
Supplement: S6 Table — (DOC) [file pone.0155270.s006.doc]

**Table S6.** Association of haplotypes and diplotypes of *SNAI1* and *TWIST1* with EC risk.

|  | | | Cases (%) | Controls (%) | | | OR (95% CI) | *P* | aOR (95% CI)^a^ | *P*^a^ |
| --- | --- | --- | --- | --- | --- | --- | --- | --- | --- | --- |
| Block of *SNAI1* (rs6125849 + rs4647959 + rs6020178) ^b^ | | | | | | | | | | |
| Haplotype | ATT | 477 (46.22) | | | 659 (46.61) | Reference | |  | Reference |  |
|  | GTT | 381 (36.92) | | | 548 (38.76) | 0.93 (0.78-1.09) | | 0.3561 | 0.90 (0.60-1.34) | 0.5998 |
|  | GCC | 83 (8.04) | | | 122 (8.63) | 0.93 (0.69-1.24) | | 0.6079 | 1.12 (0.65-1.92) | 0.6771 |
|  | GTC | 79 (7.66) | | | 83 (5.87) | 1.33 (0.97-1.83) | | 0.0804 | 1.21 (0.65-2.25) | 0.5528 |
| Diplotype | ATT-GTT | 176 (34.11) | | | 259 (36.63) | Reference | |  | Reference |  |
|  | ATT-ATT | 109 (21.12) | | | 150 (21.22) | 0.99 (0.75-1.31) | | 0.9689 | 1.00 (0.71-1.41) | 0.9835 |
|  | GTT-GTT | 74 (14.34) | | | 111 (15.70) | 0.90 (0.65-1.24) | | 0.5125 | 0.88 (0.59-1.32) | 0.5318 |
|  | ATT-GCC | 36 (6.98) | | | 61 (8.63) | 0.79 (0.52-1.22) | | 0.2921 | 0.67 (0.39-1.15) | 0.1493 |
|  | GTT-GCC | 30 (5.81) | | | 39 (5.52) | 1.06 (0.65-1.73) | | 0.8230 | 0.91 (0.47-1.79) | 0.7944 |
|  | ATT-GTC | 40 (7.75) | | | 37 (5.23) | 1.52 (0.96-2.42) | | 0.0751 | 1.14 (0.66-1.97) | 0.6300 |
| Block of *TWIST1* (rs2285682 + rs2285681) ^b^ | | | | | | | | | | |
| Haplotype | TG | 740 (71.71) | | | 1028 (72.70) | Reference | |  | Reference |  |
|  | TC | 154 (14.92) | | | 206 (14.57) | 1.03 (0.82-1.29) | | 0.8070 | 1.06 (0.76-1.47) | 0.7514 |
|  | GC | 135 (13.08) | | | 177 (12.52) | 1.05 (0.83-1.34) | | 0.6790 | 1.05 (0.76-1.45) | 0.7909 |
| Diplotype | TG-TG | 263 (50.97) | | | 371 (52.48) | Reference | |  | Reference |  |
|  | TG-TC | 119 (23.06) | | | 151 (21.36) | 1.10 (0.84-1.45) | | 0.4780 | 1.17 (0.83-1.64) | 0.3774 |
|  | TG-GC | 93 (18.02) | | | 132 (18.67) | 0.96 (0.71-1.29) | | 0.7735 | 1.04 (0.72-1.49) | 0.8423 |
|  | TC-GC | 19 (3.68) | | | 25 (3.54) | 1.04 (0.57-1.92) | | 0.8919 | 0.80 (0.38-1.70) | 0.5667 |
|  | TC-TC | 8 (1.55) | | | 15 (2.12) | 0.73 (0.31-1.73) | | 0.4695 | 0.43 (0.13-1.38) | 0.1542 |

EC, endometrial cancer; OR, odds ratios; CI, confidence intervals.

^a^ Adjusted for BMI, age at menarche, age at primiparity, [number](http://www.iciba.com/number/) [of](http://www.iciba.com/of/) childbirth, menopause status and family history of cancer in first-degree relatives.

^b^ Haplotypes and diplotypes with frequency less than 1% were omitted.
